# The Mitochondrial Genome of the Imperiled Goliath Grouper *Epinephelus itajara*: Selective Pressures in Protein Coding Genes, Secondary Structure of tRNA Genes, and Phylogenetic Placement

**DOI:** 10.1002/ece3.71795

**Published:** 2025-07-20

**Authors:** Kyla Padgett, J. Antonio Baeza

**Affiliations:** ^1^ Department of Biological Sciences Clemson University Clemson South Carolina USA; ^2^ Smithsonian Marine Station at Fort Pierce Smithsonian Institution Fort Pierce Florida USA; ^3^ Departamento de Biología Marina Universidad Catolica del Norte Coquimbo Chile

## Abstract

The goliath grouper *Epinephelus itajara* (Perciformes: Epinephelidae) is a large, critically endangered fish distributed across coastal habitats in the western Atlantic Ocean, from Florida to southern Brazil, and with additional populations in the eastern Pacific basin. Conservation concerns for this species stem from historical overfishing, habitat loss, and life‐history traits such as slow growth and late sexual maturity. In this study, to aid conservation efforts, we assembled and characterized the complete mitochondrial genome of *E. itajara* . The mitochondrial genome of *Epinephelus itajara* is 16,561 bp long and comprises 13 protein‐coding genes (PCGs), two ribosomal RNA genes (12S and 16S rRNA), 22 transfer RNA (tRNA) genes, and an 856 bp control region. Gene order is identical to that reported for other congeneric species. The overall A + T content is 56%, and codon usage shows a preference for A + T‐rich codons. All PCGs were found to be under purifying selection, with variation in selective pressure among genes; *cox1* and *nad4* were under the strongest and weakest selection, respectively. Secondary structure analysis of the tRNA genes displayed typical cloverleaf secondary structures, except for trnS1, which lacked a complete D‐arm. Comparative analyses between MiTFi and RASP2 revealed that MiTFi provided more accurate predictions of tRNA secondary structures. The control region exhibited a high A + T content (69.9%), multiple microsatellite motifs, and one tandem repeat, along with hairpin secondary structures. These features mirror findings in closely related species. A maximum likelihood phylogenetic analysis based on translated PCGs did not support the monophyletic status of the genus *Epinephelus* and indicated a sister relationship between *Epinephelus itajara* and *Epinephelus lanceolatus* , another large‐bodied grouper from the Indo‐Pacific Ocean. The newly sequenced mitochondrial genome of *Epinephelus itajara* provides a new genomic resource that can support future conservation efforts.

## Graphical abstract

This study presents the complete mitochondrial genome of the critically endangered Goliath Grouper ( *Epinephelus itajara* ), totaling 16,561 bp and comprising 13 protein‐coding genes, 22 tRNAs, and two rRNAs. Genomic analysis revealed conserved gene order, A + T‐rich codon usage, and purifying selection across all protein‐coding genes, providing valuable resources for conservation genetics and species identification. Photograph by Albert Kok, used with permission.

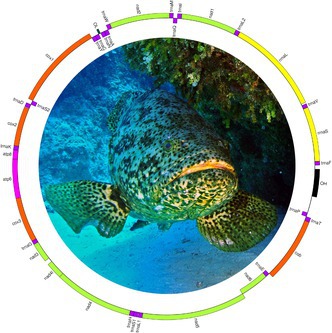

## 1 Introduction

Groupers belonging to the subfamily Epinephelinae (Perciformes: Epinephelidae) can reach large body sizes and exhibit wide mouths with which they create a strong vacuum to draw prey into their mouth, acting as highly effective predators (Sadovy and Eklund 1999; Rimmer and Glamuzina 2019). Among them, the goliath grouper, *Epinephelus itajara* , is the largest member of the family in the Atlantic Ocean, though larger species exist in other regions such as the giant grouper inhabiting the Indo‐Pacific region (Craig et al. 2024). *Epinephelus itajara* reaches lengths of over 2.4 m and weighs up to 363 kg (Bullock et al. 1992; Morris et al. 2000; Sadovy and Eklund 1999). This massive, mottled brown fish inhabits coastal waters off the Atlantic Ocean, ranging from Florida in the southeastern USA to Paraná in southern Brazil (Froese and Pauly 2024). Additionally, *Epinephelus itajara* has been reported in the eastern Pacific Ocean, with populations ranging from Costa Rica to Peru. However, genetic evidence suggests that these Pacific populations represent a distinct and separate species from the Atlantic *Epinephelus itajara* , despite looking alike morphologically (Craig et al. 2009). Small juvenile groupers are commonly found in structurally complex habitats formed by root networks of the red mangrove *Rhizophora mangle* , which serve as important nurseries by providing shelter and abundant food resources (Koenig et al. 2007). As they mature, they transition to coral reefs, where they have access to a variety of prey. These impressive predators primarily feed on crustaceans such as spiny lobsters, octopuses, shrimp, and crabs, along with other vertebrates such as numerous species of teleost fish and even young sea turtles (Artero et al. 2015). Predators of the goliath grouper include barracudas *Sphyraena* spp., the king mackerel *Scomberomorus cavalla* , moray eels (*Muraenidae* spp.), and sandbar sharks *Carcharhinus plumbeus* (Bakermans and San Martín 2022). The goliath grouper is a protogynous hermaphrodite (female to male sex changer), exhibits slow growth, and reaches sexual maturity relatively late, around 8 years of age (Murie et al. 2023; Bakermans and San Martín n.d.; Orth 2023). Goliath groupers can live for over 30 years, with the oldest recorded individual attaining 37 years old (Bullock et al. 1992).

*Epinephelus itajara* faces significant conservation challenges due to historical overfishing and habitat destruction paired with the species' slow growth and late maturity (Koenig et al. 2020), which do not permit their populations to be resilient to intense fishing practices. Goliath groupers are heavily fished, given their large body size and meat quality, which drives a very high market value for this species (Orth 2023). Coastal development and pollution have reduced shelter availability and food sources for this and other fish as well (Koenig et al. 2020). The species' life history traits limit its capacity for population recovery, rendering it highly vulnerable to the combined impacts of excessive fishing pressure and habitat loss. Goliath grouper populations in Florida declined by up to 95% of their population prior to the implementation of anti‐fishery protections in 1990, which led to the species being designated as a candidate for listing under the Endangered Species Act in 1991 and later as a species of concern (McClenachan 2009; NOAA Fisheries 2023). Today, the goliath grouper remains classified as ‘Critically Endangered’ by the International Union for Conservation of Nature (IUCN), highlighting the urgent need for comprehensive conservation efforts. These measures include fishing bans, habitat restoration, and broader ecosystem management strategies aimed at ensuring the species' long‐term survival (Bertoncini et al. 2018).

Due to the imperiled status of *Epinephelus itajara* , generating genetic and genomic resources is essential to support conservation strategies focusing on this remarkable species. Unfortunately, there is limited genomic information available for *Epinephelus itajara* , though several genetic studies have been conducted to support its conservation. Among them, a study of populations from the Northern Brazilian coast that utilized mitochondrial DNA sequences, specifically fragments of the protein‐coding gene cytochrome b (*cob*) and control region, revealed low genetic diversity in the studied populations and greater genetic variability in populations inhabiting well‐preserved mangrove forests compared to those present in degraded habitats, highlighting the importance of healthy mangrove ecosystems for the species conservation and fisheries management (Silva‐Oliveira et al. 2008). Illegal fishing remains a major threat to the conservation of *Epinephelus itajara* , jeopardizing efforts to protect and restore its population (Giglio et al. 2014). In a second study, to help prevent grouper mislabeling and curb illegal fishing, species‐specific primers were developed for the mitochondrial Cytochrome Oxidase subunit I (*cox1*) gene for the simultaneous identification of nine different species of the subfamily Epinephelidae from the western Atlantic basin using PCR (Damasceno et al. 2016). In a more recent study, DNA barcoding of the same COI gene was applied to fish market samples along the Brazilian coast, revealing that 77.3% of *Epinephelus itajara* specimens were illegally commercialized (Almeida et al. 2024). Beyond mitochondrial data, several studies have expanded the nuclear genomic toolkit for the species. Among them, the Florida Fish and Wildlife Research Institute isolated 40 microsatellite markers, developing 29 polymorphic microsatellite loci for both Atlantic and Pacific goliath groupers. These markers were validated using samples from both species and revealed significant genetic differentiation between the Atlantic and Pacific populations. The study above also noted fixed allelic differences and suggested that *Epinephelus itajara* may show signs of a genetic bottleneck, likely due to historical overharvesting. The developed microsatellite markers provide a valuable resource for future studies of population structure, gene flow, and conservation genetics in these species (Seyoum et al. 2013). Similarly, 12 novel exon‐primed intron‐crossing (EPIC) nuclear markers were developed for *Epinephelus itajara* using samples from northern Brazil (Silva‐Oliveira et al. 2013). Several of these markers revealed intraspecific polymorphism and demonstrated utility across multiple *Epinephelus* species. These markers are also valuable tools in population genetic and phylogeographic studies that are beneficial to the conservation of this species. Inter Simple Sequence Repeat (ISSR) nuclear markers can also be used in population genetics and were developed for *Epinephelus itajara* in Brazil (Benevides et al. 2014). Benevides et al. (2014) found moderate global genetic variation (~50%), but identified a genetically distinct population in Santa Catarina, Brazil, which showed the highest levels of genetic diversity and evidence of historical isolation. Their analyses revealed two evolutionarily significant units (ESUs) within *Epinephelus itajara* 's Atlantic range, suggesting limited gene flow between Santa Catarina, Brazil, and other populations. These findings emphasize the importance of region‐specific conservation strategies due to evolutionary distinctiveness. Lastly, a recent development of a simple and cost‐effective PCR‐based method was utilized to accurately identify *Epinephelus itajara* using a species‐specific primer (16S‐RYOP4) targeting a region of the mitochondrial 16S rRNA gene. The method reliably distinguished *Epinephelus itajara* from other species of *Epinephelus* and closely related genera by producing a distinctive two‐band electrophoresis pattern (423 and 621 bp). This tool is particularly useful for enforcement agencies in regions with high rates of illegal harvesting, as it allows for rapid identification of fishery products even when morphological features are removed (Oliveira et al. 2021).

In this study, we provide a new genomic resource for the goliath grouper; we sequenced the mitochondrial genome of *Epinephelus itajara* and analyzed it in detail. Following recommendations in Baeza (2022), we analyzed the nucleotide composition of the entire mitochondrial genome, codon usage of and selective pressure in the mitochondrial protein‐coding genes (PCGs), examined the secondary structure of the transfer RNA (tRNA) genes, and explored the presence of short tandem repeats and microsatellites within the control region. This genomic resource is expected to support conservation plans for this iconic fish, for instance, biomonitoring of this species using environmental DNA.

## 2 Methods

The mitochondrial genome of *E. itajara* was sequenced using DNA extracted from a specimen (USNM: FISH:416759) belonging to the fish collection of the National Museum of Natural History, Smithsonian Institution, Washington, DC, USA. This specimen was collected from the Atlantic coast of Florida, United States (Port Canaveral Main Channel, Brevard County; 28.4100° N, 80.6400° W) at a depth of 2 m. Following Cady et al. (2021) and Skufca and Baeza (2025), muscle tissue was used for extracting genomic DNA (gDNA) using the AutoGenPrep 965 automated DNA extraction robot (AutoGen, Holliston, MA, USA). Then, as in Cady et al. (2021) and Skufca and Baeza (2025), an Illumina shotgun library (paired‐end) was prepared utilizing the NEB Ultra II DNA library prep kit (New England Biolabs, Ipswich, MA, USA). Short‐read sequencing was achieved using an Illumina MiSeq (Illumina, San Diego, CA, USA) platform (at 2 × 150 cycles). Lastly, the totality of the sequenced reads (*n* = 7,927,452 pairs) was utilized for the assembly of the mitochondrial genome belonging to the goliath grouper with the pipeline GetOrganelle v. v1.7.6.1 (Jin et al. 2020).

The newly assembled mitochondrial genome was annotated using the program MITOS2 (https://usegalaxy.eu/storage, Donath et al. 2019) as implemented in the platform Galaxy Europe (https://usegalaxy.eu/login/start?redirect=None, Jalili et al. 2020). Next, the software Mega11 (Kumar et al. 2018) was used to estimate the nucleotide composition of the entire mitochondrial genome. The online web server GenomeVx (http://wolfe.ucd.ie/GenomeVx/, Conant and Wolfe 2008) was used to depict the assembled and annotated mitochondrial genome as a circular map.

Codon usage was assessed using the Codon Usage Calculator available on the web server Sequence Manipulation Suite (https://www.bioinformatics.org/sms2/codon_usage.html, Stothard 2000). EZcodon (http://ezmito.unisi.it/ezcodon, Cucini et al. 2021) was also used to calculate the relative synonymous codon usage (RSCU) of the mitochondrial protein‐coding genes.

We assessed whether PCGs in the studied mitochondrial genome were subjected to neutral, negative (purifying), or positive (diversifying) selection. To achieve this goal, first, each mitochondrial protein‐coding gene was aligned to an orthologous sequence belonging to the congeneric *Epinephelus akaara* (EU043377.1) using the program MEGA11. Next, we estimated Ka, Ks, and the Ka/Ks ratio using the program KaKs Calculator 3 (Zhang 2022) and the congeneric *Epinephelus akaara* (EU043377.1) as an outgroup. The non‐synonymous substitution rate (Ka), the synonymous substitution rate (Ks), and the ratio of Ka/Ks were used to infer the type of selection acting on a gene. A Ka/Ks ratio < 1 indicates purifying selection, a Ka/Ks ratio = 1 indicates that the locus may be evolving neutrally with respect to selection, and a Ka/Ks ratio > 1 indicates positive selection. For our calculations, we applied the MYN model to account for variability in mutation rate across the length of the studied sequences. The statistical significance of the estimated Ka/Ks ratio is assessed using a *p*‐value from a Fisher test (Zhang 2022).

The secondary structures of all mitochondrial transfer RNA (tRNA) genes were predicted using two different bioinformatic tools, that is, the program MiTFi (Jühling et al. 2012) within the MITOS2 platform and the newly released software RASP2 (http://rasp.zhanglab.net/predstr/, Mu et al. 2025). We compared the secondary structure predicted by the two tools to benchmark the accuracy of RASP2. All secondary structure predictions were visualized using the platform Forna (http://rna.tbi.univie.ac.at/forna/, Kerpedjiev et al. 2015).

In the control region of the studied mitochondrial genome, microsatellites were detected using the web server Microsatellite Repeats Finder (http://insilico.ehu.es/mini_tools/microsatellites/, Bikandi et al. 2004). Tandem repeats were also identified using the program Tandem Repeat Finder (https://tandem.bu.edu/trf/trf.html, Benson 1999). Lastly, the RNAfold Web Server (Lorenz et al. 2011) was used to predict the secondary structure of the control region. RNAfold predicted both a Minimum Free Energy (MFE) secondary structure that represents the CR's most stable conformation with the lowest free energy and a Centroid secondary structure, which evaluates all possible folds and selects the one with the lowest ensemble distance (Lorenz et al. 2011).

### 2.1 Phylogenetic Placement of the Goliath Grouper Based on Mitochondrial Genomes

We explored the phylogenetic position of the goliath grouper in the genus *Epinephelus* and examined the monophyletic status of the same genus using the phylogenetic signal provided by mitochondrial PCGs. The phylomitogenomic analysis included the mitochondrial genome of *Epinephelus itajara* (sequenced as part of BioProject PRJNA720393 and available under SRA accession SRX17832793) along with 54 additional mitochondrial genomes from 40 other *Epinephelus* species retrieved from GenBank (consulted 25 April 2025). Mitochondrial genomes belonging to the genera *Aethaloperca* (*n* = 2 sequences belonging to 1 species), *Anyperodon* (*n* = 1 sequence belonging to a single species), *Cephalopholis* (*n* = 11 sequences belonging to 10 species), *Cromileptes* (*n* = 3 sequences belonging to a single species), *Hyporthodus* (*n* = 3 sequences belonging to 3 species), *Mycteroperca* (*n* = 3 sequences belonging to 3 species), *Triso* (*n* = 1), *Plectropomus* (*n* = 6 sequences belonging to 3 species), and *Variola* (*n* = 2 sequences belonging to 2 species) belonging to the family Epinephelidae, and other species not belonging to the aforementioned family: *Etheostoma* (*n* = 1), *Caranx* (*n* = 1), *Perca* (*n* = 1), and *Toxotes* (*n* = 1), were used as outgroups during the analysis (Table S1).

To start the analysis, we aligned each mitochondrial PCG using the program ClustalW (Thompson 1997) to then remove regions in each alignment that were poorly aligned with the software GBlocks (Castresana 2000; Talavera and Castresana 2007). Next, we selected the best model of character evolution using the program ProtTest (Abascal et al. 2005; Minh et al. 2013). At last, a Maximum Likelihood (ML) phylogenetic analysis was conducted on the PCG‐partitioned dataset using the web server IQTREE v. 1.6.10 (http://iqtree.cibiv.univie.ac.at—Nguyen et al. 2015). The robustness of the ML tree topology was evaluated by performing 1000 bootstrap replications of the observed dataset.

## 3 Results & Discussion

**FIGURE 1:**
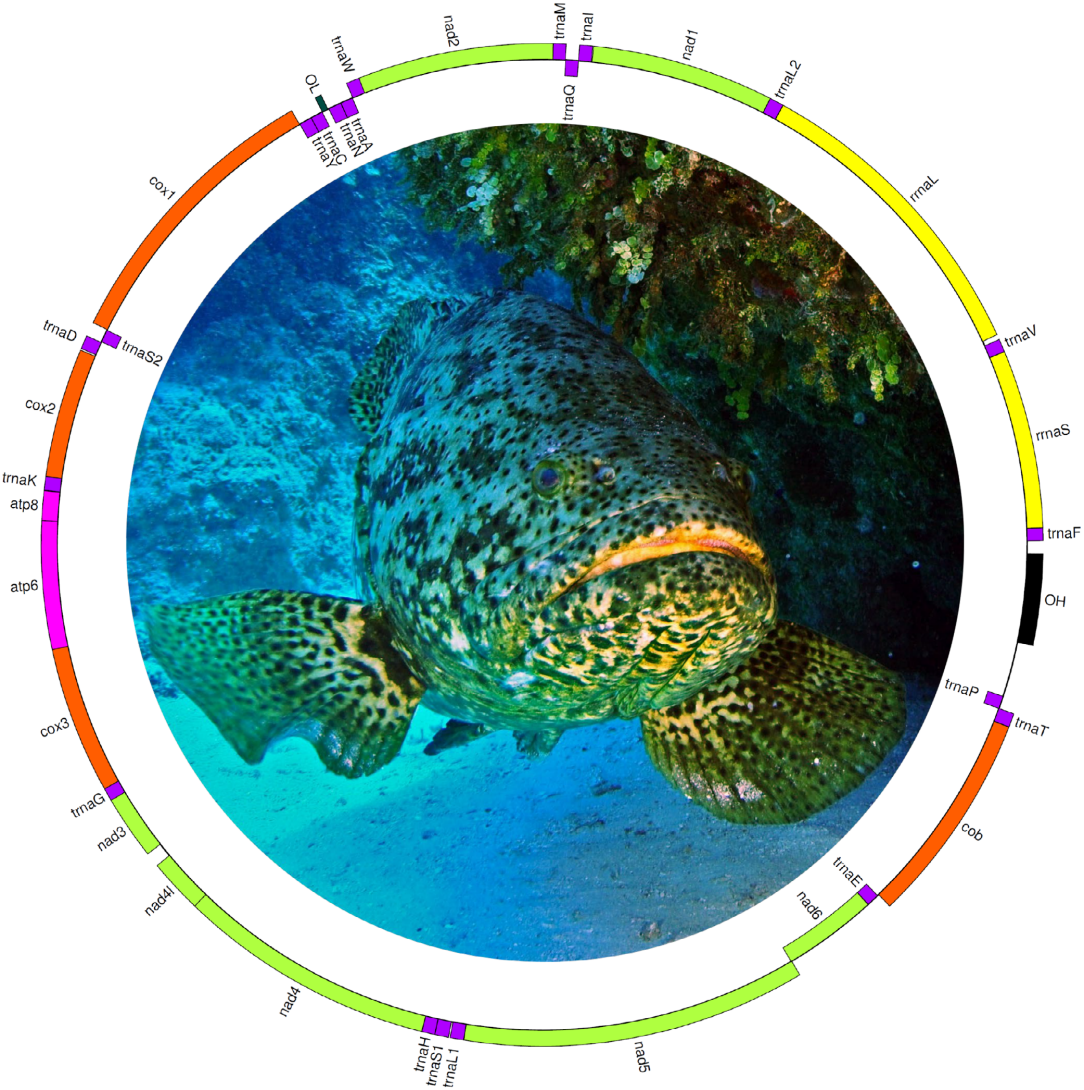
Mitochondrial genome circular map of *Epinephelus itajara* . Photograph by Albert Kok, used with permission.

The program GetOrganelle assembled a complete (circular) mitochondrial genome of *Epinephelus itajara* (GenBank accession number OP056827.1) with an average coverage of 105× per nucleotide. The mitochondrial genome of *Epinephelus itajara* was 16,561 base pairs (bp) long and contained 13 protein‐coding genes, 22 tRNA genes, and 2 rRNA genes. It also includes a non‐coding region 856 bp long (Figure 1, Table 1). Genes in the circular mitochondrial genome map were color‐coded based on their type and functional classification. Transfer RNA (tRNA) genes are shown in purple, while ribosomal RNA (rRNA) genes (12S and 16S) are highlighted in yellow. The black regions represent the non‐coding control region, including the origin of heavy and light strand replication (OH and OL). Green indicates NADH dehydrogenase subunits (nad1–nad6 and nad4L), whereas orange is used for cytochrome oxidase subunits (cox1, cox2, cox3) and cytochrome b (cob). Finally, pink marks the ATP synthase subunits (atp6 and atp8). In congeneric species, the mitochondrial genome length ranges from 16,418 bp in *Epinephelus coioides* to 16,965 bp in *Epinephelus areolatus* (Zhuang et al. 2013). In turn, in the family Epinephelidae, mitochondrial genome sizes vary more widely, ranging from 16,504 bp in *Cromileptes altivelis* to 16,767 bp in *Cephalopholis argus* (Zhuang et al. 2013). Thus, the mitochondrial genome length in *Epinephelus itajara* falls within the previously observed range in closely related species. Also, the gene order of *Epinephelus itajara* is identical to that reported in other congeneric species (e.g., in *Epinephelus akaara* and *Epinephelus* areolatus—Zhuang et al. 2013, among others).

**TABLE 1:** Mitochondrial genome of *Epinephelus itajara* .

Gene name	Type	Start	Stop	Strand	Length (bp)	Start codon	Stop codon	Anticodon	Continuity
tRNAF	tRNA	1	69	+	69			GAA	0
rRNAS	rRNA	70	1022	+	952				+1
tRNAV	tRNA	1023	1092	+	69			TAC	+1
rRNAL	rRNA	1119	2797	+	1678				+27
tRNAL2	tRNA	2798	2873	+	75			TAA	+1
NaD1	PCG	2874	3848	+	974	ATG	TAA		+1
tRNAI	tRNA	3853	3922	+	69			GAT	+5
tRNAQ	tRNA	3922	3992	−	0			TTG	0
tRNAM	tRNA	3993	4061	+	68			CAT	+1
NaD2	PCG	4062	5108	+	1046	ATG	TAG		+1
tRNAW	tRNA	5107	5177	+	70			TCA	−1
tRNAA	tRNA	5179	5247	−	68			TGC	+2
tRNAN	tRNA	5248	5320	−	72			GTT	+1
OL	Replication	5325	5359	+	34				+5
tRNAC	tRNA	5360	5427	−	67			GCA	+1
tRNAY	tRNA	5428	5498	−	70			GTA	+1
COX1	PCG	5500	7050	+	1550	GTG	TAA		+2
tRNAS2	tRNA	7053	7123	−	70			TGA	+3
tRNAD	tRNA	7127	7199	+	72			GTC	+4
COX2	PCG	7208	7898	+	690	ATG	T—		+9
tRNAK	tRNA	7899	7972	+	73				+1
ATP8	PCG	7974	8141	+	167	ATG	TAA	TTT	+2
ATP6	PCG	8132	8815	+	683	CTG	TAA		−9
COX3	PCG	8815	9600	+	785	ATG	TAA		0
tRNAG	tRNA	9600	9671	+	71			TCC	0
NaD3	PCG	9669	10,011	+	342	ATA	T—		−2
NaD4l	PCG	10,090	10,386	+	296	ATG	TAA		−79
NaD4	PCG	10,380	11,760	+	1380	ATG	T—		−6
tRNAH	tRNA	11,761	11,830	+	69			GTG	+1
tRNAS1	tRNA	11,831	11,904	+	73			GCT	+1
tRNAL1	tRNA	11,913	11,985	+	72			TAG	+9
NaD5	PCG	11,986	13,824	+	1838	ATG	TAA		+1
NaD6	PCG	13,821	14,342	−	521	ATG	TAG		−3
tRNAE	tRNA	14,343	14,412	−	69			TTC	+1
COB	PCG	14,420	15,560	+	1140	ATG	T—		+8
tRNAT	tRNA	15,561	15,634	+	73			TGT	+1
tRNAP	tRNA	15,635	15,704	−	69			TGG	+1
CR	Non‐coding	15,705	16,561	+	856				+1
OH	Replication	16,008	16,493	+	485				+304

*Note:* Arrangement and annotation. Gene name, gene type, start and end position in the mitochondrial genome, position in the leading (+) or lagging (−) strand, and start and stop codons for protein‐coding genes are shown.

The mitochondrial genome of *Epinephelus itajara* exhibits the following nucleotide composition: Adenine (A) = 29.3%, Guanine (G) = 15.2%, Cytosine (C) = 28.8%, and Thymine (T) = 26.7%, resulting in an A + T content of 56%. This AT‐rich composition aligns well with values observed in other groupers. In the genus *Epinephelus*, the A + T content ranges from 55% in *Epinephelus amblycephalus* to 56% in *Epinephelus lanceolatus* (Wang et al. 2022; Zhuang et al. 2013). Similarly, in the family Epinephelidae, A + T content remains consistently high, with values ranging from 55.2% in *Cephalopholis leopardus* to 56.1% in *Cephalopholis sonnerati* (Wang et al. 2022; Zhuang et al. 2013). In *Epinephelus itajara* and related species, the AT‐rich content of mitochondrial genomes might result from a combination of mutational biases and replication asymmetry (Tamura and Nei 1993; Perna and Kocher 1995; Nikolaou and Almirantis 2005; Uddin et al. 2020; Alvarenga et al. 2024).

**FIGURE 2:**
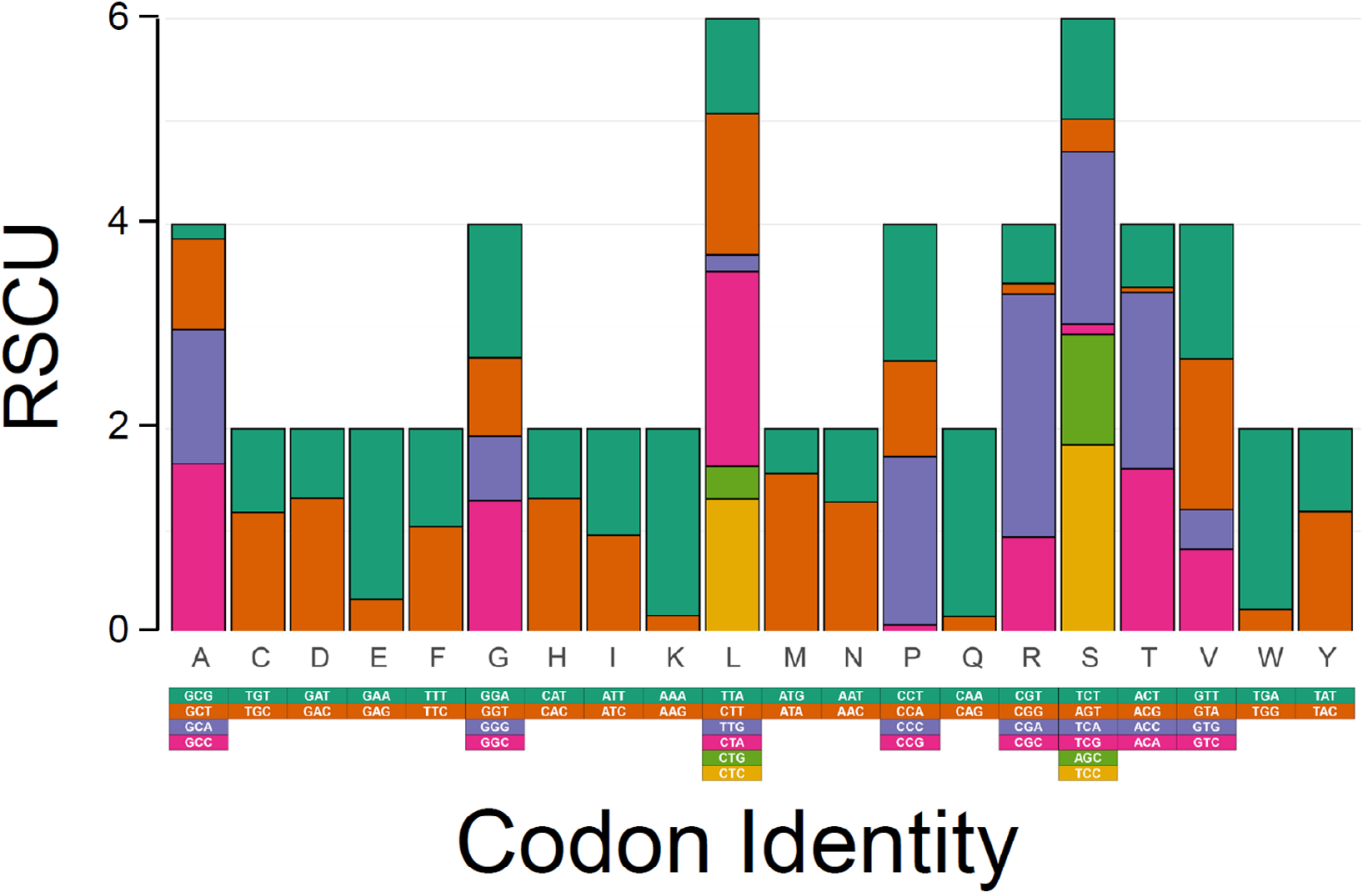
Relative synonymous codon usage (RSCU) in mitochondrial protein‐coding genes of the goliath grouper *Epinephelus itajara* .

In the PCGs of the studied mitochondrial genome, most genes possessed conventional start (ATG) and stop (TAA) codons. However, notable deviations were observed, such as the use of GTG and CTG start codons by *cox1* and *atp6*, respectively, and the presence of incomplete stop codons in *cox2*, *nad3*, *nad4*, and c*ob*. In the mitochondrial genome of *Epinephelus itajara* , PCGs do not exhibit a proportional codon usage. The most frequently used codons were CTA (Leu), CGA (Arg), AAA (Lys), and CAA (Gln). The least frequently used codons for PCGs, except stop codons, were CGG (Arg), TCG (Ser), ACG (Thr), and GCG (Ala) (Table S2). Furthermore, the RSCU analysis revealed biased usage among synonymous codons in most studied PCGs, with a preference for A + T‐rich codons over G + C‐rich codons (Figure 2). This non‐random, A + T‐rich codon usage pattern is consistent with findings in other groupers, including *Epinephelus bilobatus* , *Epinephelus maculatus* , and *Epinephelus longispinis* (He et al. 2024).

In *Epinephelus itajara* , Ka/Ks values estimated for all 13 mitochondrial PCGs were well below 1, indicating that all these genes are under purifying selective pressure (all *p*‐values < 0.05 in all cases) (Table 2). However, there were qualitative differences among genes. For example, *nad4* exhibited a notably higher Ka/Ks ratio (= 0.5669) compared to the other PCGs, suggesting it may be under relatively weaker purifying selection. In turn, *cox1* exhibited a much lower Ka/Ks ratio (= 0.0058), indicating that this gene might be under a strong purifying selection. Our results indicate that the strength of selective pressure varies among genes. Selective pressure analyses of mitochondrial PCGs have been conducted in only a few species belonging to the family Epinephelidae. Among them, all mitochondrial PCGs have been shown to experience purifying selection in *Corydoras leopardus* , *Cephalopholis spiloparaea* , *Epinephelus amblycephalus* , and *Epinephelus hexagonatus* (Wang et al. 2022).

**TABLE 2:** Selective pressure analysis of protein‐coding genes in the mitochondrial genome of *Epinephelus itajara* estimated using KaKs_Calculator.

Gene	Ka	Ks	Ka/Ks	*p*
NaD1	0.0249851	1.46574	0.0170461	1.52E‐72
NaD2	0.0706385	0.731155	0.0966122	5.26E‐38
COX1	0.0112346	1.91669	0.00586149	1.26E‐132
COX2	0.0174626	1.12656	0.0155008	1.59E‐45
ATP8	0.103113	0.952473	0.108259	3.71E‐06
ATP6	0.0482032	1.02193	0.0471687	3.42E‐42
COX3	0.0171049	0.608101	0.0281284	1.33E‐41
NaD3	0.0445345	1.17934	0.0377622	3.00E‐22
NaD4l	0.0384378	0.904612	0.0424909	2.45E‐15
NaD4	0.345154	0.608765	0.566973	0.252856
NaD5	0.0456903	1.40191	0.0325915	2.72E‐143
NaD6	0.0572596	2.17001	0.0263869	1.52E‐34
COB	0.042934	1.15065	0.037313	6.31E‐87

**FIGURE 3:**
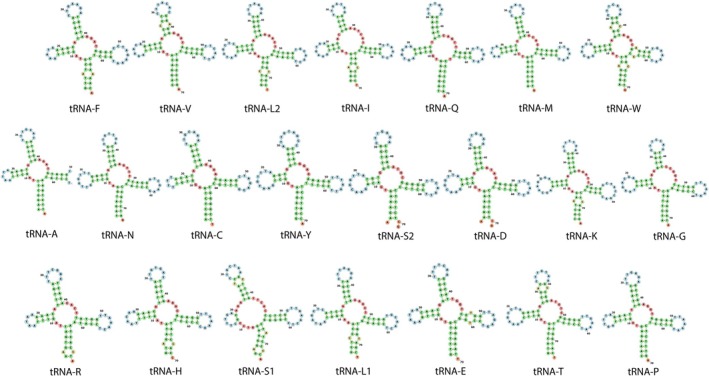
Secondary structures of the tRNA genes in the mitochondrial genome of *Epinephelus itajara* predicted by the software MiTFi as implemented in the platform MITOS2.

**FIGURE 4:**
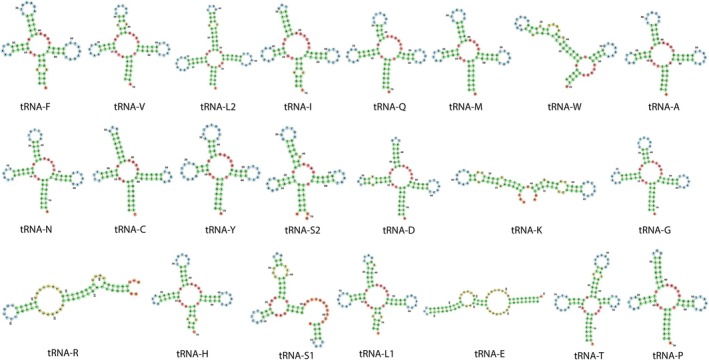
Secondary structures of the tRNA genes in the mitochondrial genome of *Epinephelus itajara* predicted by the software RASP2.

The tRNA genes in the mitochondrial genome of *Epinephelus itajara* ranged from 67 to 75 bp in length, in tRNAC and tRNAL2, respectively. The secondary structure analysis of the tRNA genes predicted by MiTFi revealed that all these genes exhibited the expected cloverleaf secondary structure except for tRNA‐S1, which lacked a complete D arm (Figure 3). This truncation is consistent with observations in the few grouper species where similar analyses have been conducted. For example, *Corydoras leopardus* and *Cephalopholis spiloparaea* also exhibit a truncated or absent D arm in their tRNA‐S1genes (Wang et al. 2022). Whether the atypical secondary structure of the tRNA‐S1gene is a conserved feature in the subfamily Epinephelinae remains to be addressed. Interestingly, major discrepancies were observed between the MiTFi and RASP secondary structure predictions (Figure 4). While MiTFi identified all 22 tRNA genes (except tRNA‐S1) with cloverleaf secondary structures, RASP2 only predicted 10 tRNA genes with a cloverleaf secondary structure, suggesting that the latter tool is not as accurate as MiTFi to predict the secondary structure of mitochondrial tRNA genes. We note that MiTFi's predictions align more closely with experimentally validated tRNA structures from related species (Wang et al. 2022).

Both the small and large ribosomal subunit RNAs in the studied mitochondrial genome were located in the leading strand. The 12S rRNA gene is approximately 952 base pairs long and is flanked by the tRNAF gene upstream (5′) and the tRNAV gene downstream (3′). The 16S rRNA gene is 1678 bp long and is flanked by the tRNAV gene upstream (5′) and the tRNAL2 gene downstream (3′). In an earlier study that described the mitochondrial genomes of 22 groupers, the 12S rRNA gene ranged from 952 to 961 bp, and the 16S rRNA gene ranged from 1695 to 1722 bp (Zhuang et al. 2013), in line with our observations. The two ribosomal genes in *Epinephelus itajara* are A + T‐rich, with the small subunit (12S rRNA) containing a 51.42% A + T content and the large subunit (16S rRNA) containing a 54.67% A + T content. This A + T‐rich composition is consistent with the results from a comparative analysis of four complete mitochondrial genomes in groupers, which reported A + T contents ranging from 53.0% to 53.6% for the 12S rRNA gene and from 55.4% to 56.9% for the 16S rRNA gene, reflecting a moderate A + T bias typical of mitochondrial rRNA genes in related species (Wang et al. 2022).

The control region (CR) of *Epinephelus itajara* is 856 bp long and includes the origin of replication of the heavy strand (OH) (Figure 1, Table 1). Notably, the origin of replication for the light strand (OL) was located outside of the CR, flanked by tRNA‐P (in the 5′ end) and tRNA‐F (3′ end). The studied CR is shorter than those found in other closely related species. For example, *E. quoyanus* has a CR of 1092 bp while the Red Grouper *Plectropomus leopardus* has a 1077 bp CR (Peng et al. 2014; Zhu and Yue 2008). Despite differences in length, these CRs share similar nucleotide composition; the control region of *E. itajara* is A + T‐rich (A + T content = 69.9%), a feature commonly observed in mitochondrial CRs across various other closely related species (Zhuang et al. 2013; Peng et al. 2014; Zhu and Yue 2008).

In the mitochondrial CR of *Epinephelus itajara* , 16 microsatellites were identified, the most common being an A + T dinucleotide motif repeated three times (Table S3). The detected microsatellites were consistently A + T‐rich, a trend also observed in related species. For example, in the Orange‐Spotted Grouper *Epinephelus coioides* , microsatellite motifs are predominantly composed of A and T nucleotides (Wang et al. 2011).

**FIGURE 5:**
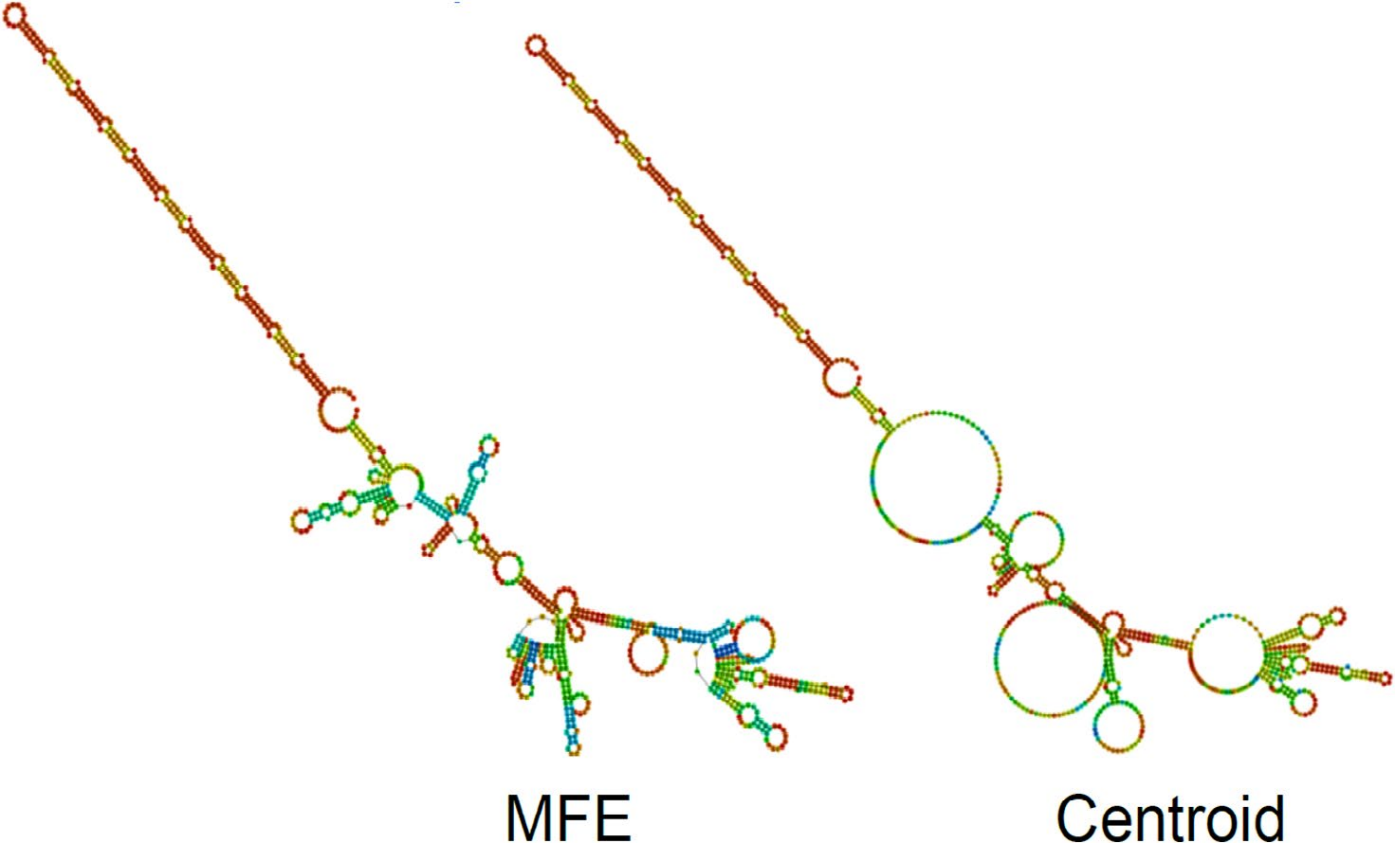
Predicted secondary structure of the control region in the mitochondrial genome of *Epinephelus itajara* . The minimum free energy (MFE) and centroid (Centroid) thermodynamic predictions by the web server RNAfold are shown.

A single tandem repeat was identified in the control region of *Epinephelus itajara* , spanning from position 1 to 205 bp of the studied region and with a period size of 17 bp (repeated 12.1 times). The consensus sequence of this repeat, 5′‐AAT TAC ATA TAT GCA TT‐3′, is A + T‐rich (A + T content = 81%, Table S4). A similar tandem repeat was observed in the congeneric Rock Grouper *Epinephelus fasciatomaculosus* , where a 141 bp long repeat was found within a 980 bp long control region (Li et al. 2013). The predicted minimum free energy (MFE) and Centroid secondary structure of **Epinephelus* itajara's* mitochondrial control region contained multiple hairpin formations, spanning across the entire region (Figure 5). Studies examining the secondary structure of the CR in other groupers are only a few, but our observations are consistent with findings in other congeneric groupers, including *Epinephelus malabaricus* , whose CR secondary structure exhibits an array of hairpin structures (Athira et al. 2022). Overall, although studies describing the mitochondrial CR are limited in the genus *Epinephelus*, in vertebrates, including co‐familial species, the control region frequently exhibits microsatellites, short tandem repeats, and hairpin loops (Terencio et al. 2013; Pereira et al. 2008; Cady et al. 2021).

### 3.1 Phylogenetic Placement of the Goliath Grouper Based on Mitochondrial Genomes

**FIGURE 6:**
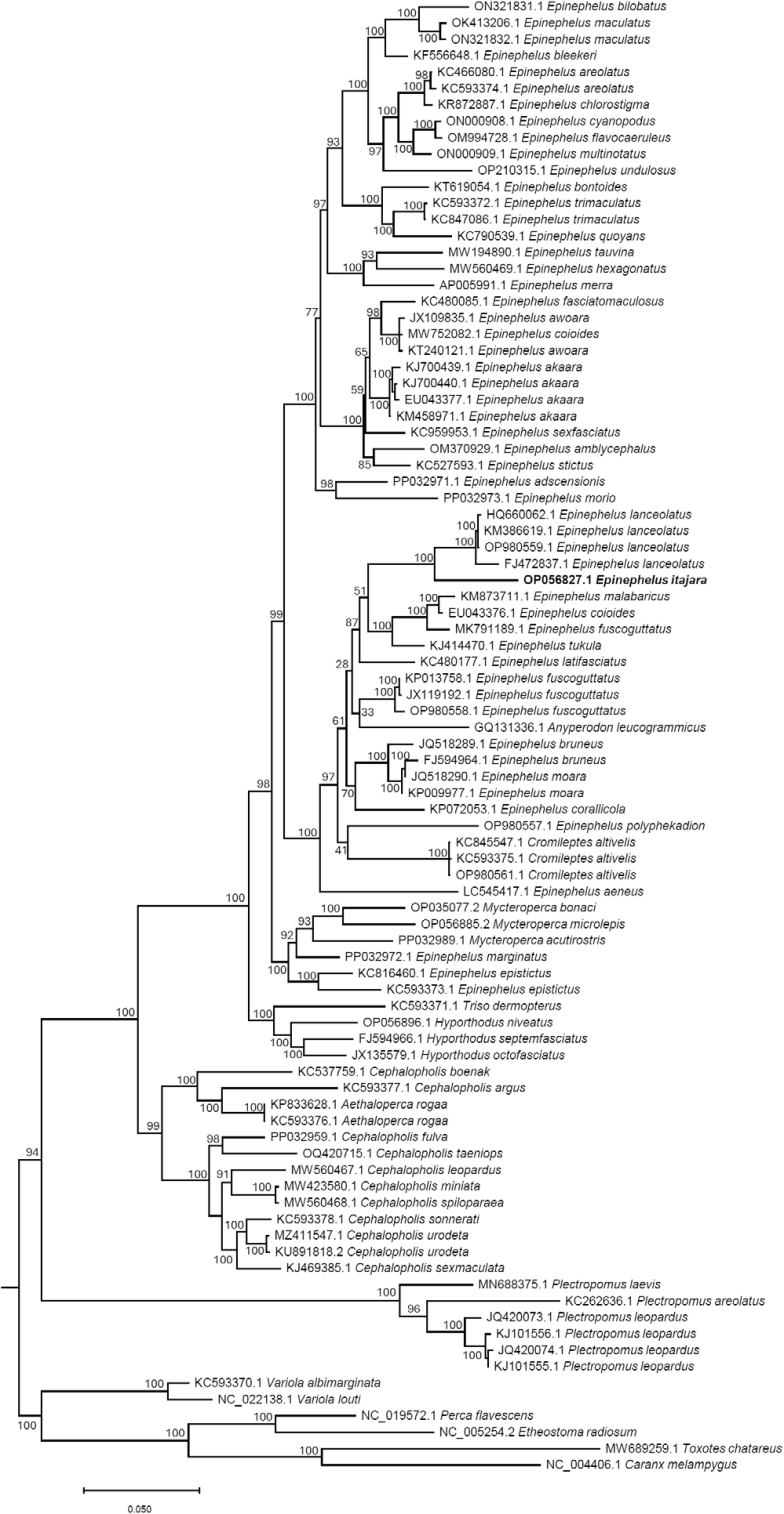
Phylomitogenomic tree generated using maximum likelihood inference to examine the placement of the goliath grouper in the genus *Epinephelus*. The tree is based on an alignment of amino acids from the 13 mitochondrial protein‐coding genes. Numbers are the Genbank accession numbers for the sequences used.

The ML phylogenetic analysis (90 terminal nodes, 3798 amino acids, and 982 informative sites) indicated that the genus *Epinephelus* was not monophyletic given the position of three genera, *Anyperodon* (*n* = 1 species), *Cromileptes* (*n* = 3 species), and *Mycteroperca* (*n* = 3 species), that clustered deep within a clade comprised of all species of *Epinephelus* used in our analysis (Figure 6). Within a well‐supported clade (bootstrap value [bv] = 98) that included the genera *Anyperodon*, *Cromileptes*, *Epinephelus*, and *Mycteroperca*, the goliath grouper occupied a derived (late branching) position, being fully supported (bv = 100) as the sister taxon to the Giant Grouper *Epinephelus lanceolatus* from the Indo‐Pacific basin. In turn, *Epinephelus itajara* and *Epinephelus lanceolatus* belonged to a poorly supported clade (bootstrap value [bv] = 51) that also included 4 other Indo‐Pacific groupers: *Epinephelus coioides* , *Epinephelus fuscoguttatus* , *Epinephelus malabaricus* , and *Epinephelus tukula* (Figure 6). Additional studies examining the phylogenetic relationships among species in the genus *Epinephelus* and closely related genera are needed to understand the evolutionary history of this remarkable clade of fish and solve taxonomic classification issues pinpointed by this analysis. Our results are in line with other mitogenome analyses, including a study that recently observed non‐monophyly in *Epinephelus* and noted that *Anyperodon* and *Cromileptes* were nested within it (Wang et al. 2022). An older study highlighted the same findings, using 16S rRNA sequences to show that *Epinephelus*, *Cephalopholis*, and *Mycteroperca* do not form distinct, monophyletic groups (Craig et al. 2001). What is new in our analysis is the inclusion of more recently sequenced mitogenomes, such as that of *Epinephelus itajara* , and a broader sampling of Indo‐Pacific species. Together, these data provide a clearer picture of the goliath grouper's evolutionary relationships and further support the need to revise the current classification of Epinephelidae.

## 4 Conclusion

We analyzed the complete mitochondrial genome of the goliath grouper, *Epinephelus itajara* , useful for the conservation of this endangered species. The gene order of the newly assembled mitochondrial genome was identical to that reported for other congeneric species. The mitochondrial genome was A + T‐rich, codons used more frequently were also A + T‐rich, and we provide evidence of purifying selection acting across all protein‐coding genes. Secondary structure predictions for tRNA genes indicated that all of them exhibited a cloverleaf secondary structure with the exception of trnS1, which lacked a full D arm, a typical feature observed in other members of the subfamily Epinephelinae. The CR was shorter than those in other closely related species but exhibited typical attributes, including A + T‐rich microsatellites, a tandem repeat, and multiple hairpins after prediction of the secondary structure of this region. These findings are consistent with structural features documented in the CR of closely related species. A maximum likelihood phylogenetic analysis supports the utility of PCGs in revealing evolutionary relationships among groupers. This new genomic resource is expected to support conservation efforts in the goliath grouper, *Epinephelus itajara* .

## Author Contributions

**Kyla Padgett:** data curation (equal), formal analysis (equal), investigation (equal), methodology (equal), project administration (equal), resources (equal), writing – original draft (equal), writing – review and editing (equal). **J. Antonio Baeza:** data curation (equal), investigation (equal), methodology (equal), project administration (equal), resources (equal), supervision (equal), validation (equal), visualization (equal), writing – original draft (equal), writing – review and editing (equal).

## Conflicts of Interest

The authors declare no conflicts of interest.

## Supporting information


Data S1.

## Data Availability

Mitochondrial genome assembly data are available online on NCBI GenBank under the accession number OP056827.1.

## References

[ece371795-bib-0061] Abascal, F. , R. Zardoya , and D. Posada . 2005. “ProtTest: Selection of Best‐Fit Models of Protein Evolution.” Bioinformatics 21, no. 9: 2104–2105. 10.1093/bioinformatics/bti263. 15647292

[ece371795-bib-0001] Almeida, L. L. , M. Hostim‐Silva , M. V. Condini , et al. 2024. “Mislabeling, Illegal Capture, and Commercialization of Atlantic Goliath Grouper (*Epinephelus itajara*) on the Brazilian Coast Using DNA Barcoding.” Neotropical Ichthyology 22: e230099. 10.1590/1982-0224-2023-0099.

[ece371795-bib-0002] Alvarenga, M. , A. K. P. D'Elia , G. Rocha , et al. 2024. “Mitochondrial Genome Structure and Composition in 70 Fishes: A Key Resource for Fisheries Management in the South Atlantic.” BMC Genomics 25, no. 1: 215. 38413941 10.1186/s12864-024-10035-5 PMC10898094

[ece371795-bib-0003] Artero, C. , C. C. Koenig , P. Richard , et al. 2015. “Ontogenetic Dietary and Habitat Shifts in Goliath Grouper *Epinephelus itajara* From French Guiana.” Endangered Species Research 27, no. 2: 155–168. 10.3354/esr00661.

[ece371795-bib-0004] Athira, P. P. , M. V. Anju , V. V. Anooja , et al. 2022. “Molecular Identification and Functional Characterisation of Epinecidin From Malabar Grouper, *Epinephelus malabaricus* .” Thalassas: An International Journal of Marine Sciences 38, no. 2: 735–744.

[ece371795-bib-0057] Baeza, J. A. 2022. “Mitochondrial Genomes Assembled From Non‐Invasive eDNA Metagenomic Scat Samples in the Endangered Amur tiger *Panthera tigris altaica* .” PeerJ 10: e14428. 10.7717/peerj.14428. 36523460 PMC9745948

[ece371795-bib-0005] Bakermans, M. , and W. San Martín . 2022. “Misunderstood ‘Gentle’ Giants—The Goliath Grouper.” *Extinction Stories*. https://pressbooks.pub/extinctionstories/chapter/goliath‐grouper/.

[ece371795-bib-0064] Bakermans, M. , and W. San Martín . “Misunderstood “Gentle” Giants–The Goliath Grouper.” Extinction Stories. https://pressbooks.pub/extinctionstories/chapter/goliath‐grouper/.

[ece371795-bib-0006] Benevides, E. A. , M. N. S. Vallinoto , A. F. H. Fetter Filho , et al. 2014. “When Physical Oceanography Meets Population Genetics: The Case Study of the Genetic/Evolutionary Discontinuity in the Endangered Goliath Grouper ( *Epinephelus itajara* ; Perciformes: Epinephelidae) With Comments on the Conservation of the Species.” Biochemical Systematics and Ecology 56: 255–266. 10.1016/j.bse.2014.06.004.

[ece371795-bib-0007] Benson, G. 1999. “Tandem Repeats Finder: A Program to Analyze DNA Sequences.” Nucleic Acids Research 27, no. 2: 573–580. 10.1093/nar/27.2.573. 9862982 PMC148217

[ece371795-bib-0008] Bertoncini, A. A. , A. Aguilar‐Perera , J. Barreiros , M. T. Craig , B. Ferreira , and C. Koenig . 2018. “*Epinephelus itajara*. The IUCN Red List of Threatened Species.” 10.2305/IUCN.UK.2018-2.RLTS.T195409A46957794.en.

[ece371795-bib-0009] Bikandi, J. , R. San Millán , A. Rementeria , and J. Garaizar . 2004. “In Silico Analysis of Complete Bacterial Genomes: PCR, AFLP–PCR and Endonuclease Restriction.” Bioinformatics 20, no. 5: 798–799. 10.1093/bioinformatics/btg491. 14752001

[ece371795-bib-0010] Bullock, L. H. , M. D. Murphy , M. F. Godcharles , and M. E. Mitchell . 1992. “Age, Growth, and Reproduction of Jewfish *Epinephelus itajara* in the Eastern Gulf of Mexico.” Fishery Bulletin 90, no. 2: 243–249.

[ece371795-bib-0011] Cady, T. , K. E. Bemis , and J. A. Baeza . 2021. “The Mitochondrial Genome of the Endangered Spiny Butterfly Ray *Gymnura altavela* (Linnaeus 1758) (Myliobatiformes: Gymnuridae) Provides Insights Into Cryptic Lineages.” Mitochondrial DNA Part A DNA Mapping, Sequencing, and Analysis 32, no. 5–8: 186–194. 10.1080/24701394.2023.2251577. 37668057

[ece371795-bib-0063] Castresana, J. 2000. “Selection of Conserved Blocks from Multiple Alignments for Their Use in Phylogenetic Analysis.” Molecular Biology and Evolution 17, no. 4: 540–552. 10.1093/oxfordjournals.molbev.a026334. 10742046

[ece371795-bib-0012] Conant, G. C. , and K. H. Wolfe . 2008. “GenomeVx: Simple Web‐Based Creation of Editable Circular Chromosome Maps.” Bioinformatics 24, no. 6: 861–862. 10.1093/bioinformatics/btm598. 18227121

[ece371795-bib-0013] Craig, M. T. , Y. J. de Sadovy Mitcheson , and P. C. Heemstra . 2024. Groupers of the World: A Field and Market Guide. CRC Press.

[ece371795-bib-0014] Craig, M. T. , R. T. Graham , R. A. Torres , et al. 2009. “How Many Species of Goliath Grouper Are There? Cryptic Genetic Divergence in a Threatened Marine Fish and the Resurrection of a Geopolitical Species.” Endangered Species Research 7, no. 3: 167–174. 10.3354/esr00117.

[ece371795-bib-0015] Craig, M. T. , D. J. Pondella II , J. P. C. Franck , and J. C. Hafner . 2001. “On the Status of the Serranid Fish Genus Epinephelus: Evidence for Paraphyly Based Upon 16S rDNA Sequence.” Molecular Phylogenetics and Evolution 19, no. 1: 121–130. 10.1006/mpev.2000.0913. 11286497

[ece371795-bib-0016] Cucini, C. , C. Leo , N. Iannotti , et al. 2021. “EZmito: A Simple and Fast Tool for Multiple Mitogenome Analyses.” Mitochondrial DNA Part B Resources 6, no. 3: 1101–1109. 10.1080/23802359.2021.1899865. 33796755 PMC7995877

[ece371795-bib-0017] Damasceno, J. S. , R. Siccha‐Ramirez , C. Oliveira , et al. 2016. “Molecular Identification of Atlantic Goliath Grouper *Epinephelus itajara* (Lichtenstein, 1822) (Perciformes: Epinephelidae) and Related Commercial Species Applying Multiplex PCR.” Neotropical Ichthyology 14, no. 3: e150128. 10.1590/1982-0224-20150128.

[ece371795-bib-0018] Donath, A. , F. Jühling , M. Al‐Arab , et al. 2019. “Improved Annotation of Protein‐Coding Genes Boundaries in Metazoan Mitochondrial Genomes.” Nucleic Acids Research 47, no. 20: 10543–10552. 10.1093/nar/gkz833. 31584075 PMC6847864

[ece371795-bib-0019] Froese, R. , and D. Pauly . 2024. “*Epinephelus itajara* Summary Page.” https://www.fishbase.org/summary/Epinephelus‐itajara.html.

[ece371795-bib-0020] Giglio, V. J. , Á. A. Bertoncini , B. P. Ferreira , M. Hostim‐Silva , and M. O. Freitas . 2014. “Landings of Goliath Grouper, *Epinephelus itajara* , in Brazil: Despite Prohibited Over Ten Years, Fishing Continues.” Natureza & Conservação 12, no. 2: 118–123. 10.1016/j.ncon.2014.09.004.

[ece371795-bib-0021] He, H. , Z. Gao , Z. Hu , et al. 2024. “Comparative Characterization and Phylogenetic Analysis of Complete Mitogenome of Three Taxonomic Confused Groupers and the Insight to the Novel Gene Tandem Duplication in Epinephelus.” Frontiers in Marine Science 11: 1450003. 10.3389/fmars.2024.1450003.

[ece371795-bib-0022] Jalili, V. , E. Afgan , Q. Gu , et al. 2020. “The Galaxy Platform for Accessible, Reproducible and Collaborative Biomedical Analyses: 2020 Update.” Nucleic Acids Research 48, no. W1: W395–W402. 10.1093/nar/gkaa434. 32479607 PMC7319590

[ece371795-bib-0023] Jin, J.‐J. , W.‐B. Yu , J.‐B. Yang , et al. 2020. “GetOrganelle: A Fast and Versatile Toolkit for Accurate de Novo Assembly of Organelle Genomes.” Genome Biology 21: 1–31. 10.1186/s13059-020-02154-5 PMC7488116 32912315

[ece371795-bib-0024] Jühling, F. , J. Pütz , M. Bernt , et al. 2012. “Improved Systematic tRNA Gene Annotation Allows New Insights Into the Evolution of Mitochondrial tRNA Structures and Into the Mechanisms of Mitochondrial Genome Rearrangements.” Nucleic Acids Research 40, no. 7: 2833–2845. 10.1093/nar/gkr1131. 22139921 PMC3326299

[ece371795-bib-0025] Kerpedjiev, P. , S. Hammer , and I. L. Hofacker . 2015. “Forna (Force‐Directed RNA): Simple and Effective Online RNA Secondary Structure Diagrams.” Bioinformatics 31, no. 20: 3377–3379. 10.1093/bioinformatics/btv372. 26099263 PMC4595900

[ece371795-bib-0026] Koenig, C. C. , F. C. Coleman , A.‐M. Eklund , J. Schull , and J. Ueland . 2007. “Mangroves as Essential Nursery Habitat for Goliath Grouper (*Epinephelus itajara*).” Bulletin of Marine Science 80, no. 3: 567–585.

[ece371795-bib-0027] Koenig, C. C. , F. C. Coleman , and C. R. Malinowski . 2020. “Atlantic Goliath Grouper of Florida: To Fish or Not to Fish.” Fisheries 45, no. 1: 20–32. 10.1002/fsh.10349.

[ece371795-bib-0028] Kumar, S. , G. Stecher , M. Li , C. Knyaz , and K. Tamura . 2018. “MEGA X: Molecular Evolutionary Genetics Analysis Across Computing Platforms.” Molecular Biology and Evolution 35, no. 6: 1547–1549. 10.1093/molbev/msy096. 29722887 PMC5967553

[ece371795-bib-0029] Li, J.‐L. , M. Liu , and Y.‐Y. Wang . 2013. “Complete Mitochondrial Genome of the Rock Grouper *Epinephelus fasciatomaculosus* (Pisces: Perciformes).” Mitochondrial DNA 24, no. 6: 625–626. 23521138 10.3109/19401736.2013.772156

[ece371795-bib-0030] Lorenz, R. , S. H. Bernhart , C. Höner zu Siederdissen , et al. 2011. “ViennaRNA Package 2.0.” Algorithms for Molecular Biology 6: 1–14. 10.3109/19401736.2013.772156. 22115189 PMC3319429

[ece371795-bib-0031] McClenachan, L. 2009. “Historical Declines of Goliath Grouper Populations in South Florida, USA.” Endangered Species Research 7, no. 3: 175–181. 10.3354/esr00167.

[ece371795-bib-0062] Minh, B. Q. , M. A. T. Nguyen , and A. von Haeseler . 2013. “Ultrafast Approximation for Phylogenetic Bootstrap.” Molecular Biology and Evolution 30, no. 5: 1188–1195. 10.1093/molbev/mst024. 23418397 PMC3670741

[ece371795-bib-0032] Morris, A. V. , C. M. Roberts , and J. P. Hawkins . 2000. “The Threatened Status of Groupers (Epinephelinae).” Biodiversity and Conservation 9: 919–942.

[ece371795-bib-0033] Mu, K. , Y. Fei , Y. Xu , and Q. C. Zhang . 2025. “RASP v2. 0: An Updated Atlas for RNA Structure Probing Data.” Nucleic Acids Research 53, no. D1: D211–D219. 10.1093/nar/gkae1117. 39546630 PMC11701657

[ece371795-bib-0034] Murie, D. J. , D. C. Parkyn , C. C. Koenig , et al. 2023. “Age, Growth, and Functional Gonochorism With a Twist of Diandric Protogyny in Goliath Grouper From the Atlantic Coast of Florida.” Fishes 8, no. 8: 412. 10.3390/fishes8080412.

[ece371795-bib-0056] Nguyen, L.‐T. , H. A. Schmidt , A. von Haeseler , and B. Q. Minh . 2015. “IQ‐TREE: A Fast and Effective Stochastic Algorithm for Estimating Maximum‐Likelihood Phylogenies.” Molecular Biology and Evolution 32, no. 1: 268–274. 10.1093/molbev/msu300. 25371430 PMC4271533

[ece371795-bib-0035] Nikolaou, C. , and Y. Almirantis . 2005. “A Study on the Correlation of Nucleotide Skews and the Positioning of the Origin of Replication: Different Modes of Replication in Bacterial Species.” Nucleic Acids Research 33, no. 21: 6816–6822. 10.1093/nar/gki988. 16321966 PMC1301597

[ece371795-bib-0036] NOAA Fisheries . 2023. Atlantic Goliath Grouper—Management. National Oceanic and Atmospheric Administration. https://www.fisheries.noaa.gov/species/atlantic‐goliath‐grouper/management.

[ece371795-bib-0037] Oliveira, Y. , R. Alencar , Y. Oliveira , et al. 2021. “Simple and Safe Approach for Molecular Identification of the Endangered Species *Epinephelus itajara* .” Conservation Genetics Resources 13, no. 2: 127–130. 10.1007/s12686-021-01195-7.

[ece371795-bib-0038] Orth, D. J. 2023. “Grouper and Spawning Aggregations.” Fish, Fishing, and Conservation. 10.21061/fishandconservation.

[ece371795-bib-0039] Peng, Z. , J. Chen , T. Lai , Y. Huang , and L. Wu . 2014. “Complete Mitochondrial Genome of the Longfin Grouper *Epinephelus quoyanus* (Serranidae: Epinephelinae).” Mitochondrial DNA 25: 175–176. 10.3109/19401736.2013.792063. 23631368

[ece371795-bib-0040] Pereira, F. , P. Soares , J. Carneiro , et al. 2008. “Evidence for Variable Selective Pressures at a Large Secondary Structure of the Human Mitochondrial DNA Control Region.” Molecular Biology and Evolution 25, no. 12: 2759–2770. 10.1093/molbev/msn225. 18845547

[ece371795-bib-0041] Perna, N. T. , and T. D. Kocher . 1995. “Patterns of Nucleotide Composition at Fourfold Degenerate Sites of Animal Mitochondrial Genomes.” Journal of Molecular Evolution 41: 353–358. 7563121 10.1007/BF00186547

[ece371795-bib-0042] Rimmer, M. A. , and B. Glamuzina . 2019. “A Review of Grouper (Family Serranidae: Subfamily Epinephelinae) Aquaculture From a Sustainability Science Perspective.” Reviews in Aquaculture 11, no. 1: 58–87. 10.1111/raq.12226.

[ece371795-bib-0043] Sadovy, Y. , and A.‐M. Eklund . 1999. “Synopsis of Biological Data on the Nassau Grouper, *Epinephelus striatus* (Bloch, 1792), and the Jewfish, *E. itajara* (Lichtenstein, 1822).”

[ece371795-bib-0044] Seyoum, S. , M. D. Tringali , B. L. Barthel , et al. 2013. “Isolation and Characterization of 29 Polymorphic Microsatellite Markers for the Endangered Atlantic Goliath Grouper ( *Epinephelus itajara* ), and the Pacific Goliath Grouper ( *E. quinquefasciatus* ).” Conservation Genetics Resources 5: 729–732. 10.1007/s12686-013-9892-x.

[ece371795-bib-0045] Silva‐Oliveira, G. C. , P. S. D. Rêgo , H. Schneider , I. Sampaio , and M. Vallinoto . 2008. “Genetic Characterisation of Populations of the Critically Endangered Goliath Grouper (*Epinephelus itajara*, Serranidae) From the Northern Brazilian Coast Through Analyses of mtDNA.” Genetics and Molecular Biology 31: 988–995. 10.1590/S1415-47572008005000016.

[ece371795-bib-0046] Silva‐Oliveira, G. C. , A. B. C. Silva , Y. Oliveira , et al. 2013. “New Nuclear Primers for Molecular Studies of Epinephelidae Fishes.” Conservation Genetics Resources 5: 165–168. 10.1007/s12686-012-9759-6.

[ece371795-bib-0058] Skufca, K. , and J. A. Baeza . 2025. “The Complete Mitochondrial Genome of *Squalus cubensis* and Comparative Mitogenomics and Phylomitogenomics of the Family Squalidae.” Ecology and Evolution 15, no. 5: e71412. 10.1002/ece3.71412. 40336546 PMC12055450

[ece371795-bib-0047] Stothard, P. 2000. “The Sequence Manipulation Suite: JavaScript Programs for Analyzing and Formatting Protein and DNA Sequences.” BioTechniques 28: 1102–1104. 10.2144/00286ir01. 10868275

[ece371795-bib-0060] Talavera, G. , and J. Castresana . 2007. “Improvement of Phylogenies After Removing Divergent and Ambiguously Aligned Blocks from Protein Sequence Alignments.” Systematic Biology 56, no. 4: 564–577. 10.1080/10635150701472164. 17654362

[ece371795-bib-0048] Tamura, K. , and M. Nei . 1993. “Estimation of the Number of Nucleotide Substitutions in the Control Region of Mitochondrial DNA in Humans and Chimpanzees.” Molecular Biology and Evolution 10, no. 3: 512–526. 10.1093/oxfordjournals.molbev.a040023. 8336541

[ece371795-bib-0049] Terencio, M. L. , C. H. Schneider , M. C. Gross , E. Feldberg , and J. I. R. Porto . 2013. “Structure and Organization of the Mitochondrial DNA Control Region With Tandemly Repeated Sequence in the Amazon Ornamental Fish.” Mitochondrial DNA 24, no. 1: 74–82. 10.3109/19401736.2012.717934. 22954310

[ece371795-bib-0059] Thompson, J. 1997. “The CLUSTAL_X Windows Interface: Flexible Strategies for Multiple Sequence Alignment Aided by Quality Analysis Tools.” Nucleic Acids Research 25, no. 24: 4876–4882. 10.1093/nar/25.24.4876. 9396791 PMC147148

[ece371795-bib-0050] Uddin, A. , T. H. Mazumder , P. A. Barbhuiya , and S. Chakraborty . 2020. “Similarities and Dissimilarities of Codon Usage in Mitochondrial ATP Genes Among Fishes, Aves, and Mammals.” IUBMB Life 72, no. 5: 899–914. 10.1002/iub.2231. 31958218

[ece371795-bib-0051] Wang, C. , P. Ye , M. Liu , et al. 2022. “Comparative Analysis of Four Complete Mitochondrial Genomes of Epinephelidae (Perciformes).” Genes 13, no. 4: 660. 10.3390/genes13040660. 35456466 PMC9029768

[ece371795-bib-0052] Wang, L. , Z. Meng , X. Liu , Y. Zhang , and H. Lin . 2011. “Genetic Diversity and Differentiation of the Orange‐Spotted Grouper ( *Epinephelus coioides* ) Between and Within Cultured Stocks and Wild Populations Inferred From Microsatellite DNA Analysis.” International Journal of Molecular Sciences 12, no. 7: 4378–4394. 10.3390/ijms12074378. 21845084 PMC3155357

[ece371795-bib-0053] Zhang, Z. 2022. “KaKs_Calculator 3.0: Calculating Selective Pressure on Coding and Non‐Coding Sequences.” Genomics, Proteomics & Bioinformatics 20, no. 3: 536–540. 10.1016/j.gpb.2021.12.002. PMC9801026 34990803

[ece371795-bib-0054] Zhu, Z. Y. , and G. H. Yue . 2008. “The Complete Mitochondrial Genome of Red Grouper *Plectropomus leopardus* and Its Applications in Identification of Grouper Species.” Aquaculture 276, no. 1–4: 44–49. 10.1016/j.aquaculture.2008.02.008.

[ece371795-bib-0055] Zhuang, X. , M. Qu , X. Zhang , and S. Ding . 2013. “A Comprehensive Description and Evolutionary Analysis of 22 Grouper (Perciformes, Epinephelidae) Mitochondrial Genomes With Emphasis on Two Novel Genome Organizations.” PLoS One 8, no. 8: e73561. 10.1371/journal.pone.0073561. 23951357 PMC3739747

