# Supplementary material for: The Mitochondrial Genome of the Imperiled Goliath Grouper Epinephelus itajara: Selective Pressures in Protein Coding Genes, Secondary Structure of tRNA Genes, and Phylogenetic Placement
Source: Ecol Evol. 2025 Jul 20;15(7):e71795. doi: 10.1002/ece3.71795 (PMC12277541; doi:10.1002/ece3.71795)

**The mitochondrial genome of the imperiled goliath grouper *Epinephelus itajara*:**

**selective pressures in protein coding genes, secondary structure of tRNA genes,**

**and phylogenetic placement**

**Kyla Padgett**^a^  **and J. Antonio Baeza**^a,b,c, *^

^a^ Department of Biological Sciences, Clemson University, Clemson, SC, USA

^b^ Smithsonian Marine Station at Fort Pierce, Smithsonian Institution, Fort Pierce, FL, USA

^c^ Departamento de Biología Marina, Universidad Catolica del Norte, Coquimbo, Chile

* Corresponding author. J. Antonio Baeza. Email: baeza.antonio@gmail.com

**Supplementary Materials**

Table S1. Species Used for Phylogenetic Placement of *Epinephelus itajara.*

| **Genebank** | **Species** |
| --- | --- |
| ON321831 | *Epinephelus bilobatus* |
| OK413206 | *Epinephelus maculatus* |
| ON321832 | *Epinephelus maculatus* |
| KF556648 | *Epinephelus bleekeri* |
| KC466080 | *Epinephelus areolatus* |
| KC593374 | *Epinephelus areolatus* |
| KR872887 | *Epinephelus chlorostigma* |
| ON000908 | *Epinephelus cyanopodus* |
| OM994728 | *Epinephelus flavocaeruleus* |
| ON000909 | *Epinephelus multinotatus* |
| OP210315 | *Epinephelus undulosus* |
| KT619054 | *Epinephelus bontoides* |
| KC593372 | *Epinephelus trimaculatus* |
| KC847086 | *Epinephelus trimaculatus* |
| KC790539 | *Epinephelus quoyans* |
| MW194890 | *Epinephelus tauvina* |
| MW560469 | *Epinephelus hexagonatus* |
| AP005991 | *Epinephelus merra* |
| KC480085 | *Epinephelus fasciatomaculosus* |
| JX109835 | *Epinephelus awoara* |
| MW752082 | *Epinephelus coioides* |
| KT240121 | *Epinephelus awoara* |
| KJ700439 | *Epinephelus akaara* |
| KJ700440 | *Epinephelus akaara* |
| EU043377 | *Epinephelus akaara* |
| KM458971 | *Epinephelus akaara* |
| KC959953 | *Epinephelus sexfasciatus* |
| OM370929 | *Epinephelus amblycephalus* |
| KC527593 | *Epinephelus stictus* |
| PP032971 | *Epinephelus adscensionis* |
| PP032973 | *Epinephelus morio* |
| HQ660062 | *Epinephelus lanceolatus* |
| KM386619 | *Epinephelus lanceolatus* |
| OP980559 | *Epinephelus lanceolatus* |
| FJ472837 | *Epinephelus lanceolatus* |
| OP056827 | *Epinephelus itajara* |
| KM873711 | *Epinephelus malabaricus* |
| EU043376 | *Epinephelus coioides* |
| MK791189 | *Epinephelus fuscoguttatus* |
| KJ414470 | *Epinephelus tukula* |
| KC480177 | *Epinephelus latifasciatus* |
| KP013758 | *Epinephelus fuscoguttatus* |
| JX119192 | *Epinephelus fuscoguttatus* |
| OP980558 | *Epinephelus fuscoguttatus* |
| GQ131336 | *Anyperodon leucogrammicus* |
| JQ518289 | *Epinephelus bruneus* |
| FJ594964 | *Epinephelus bruneus* |
| JQ518290 | *Epinephelus moara* |
| KP009977 | *Epinephelus moara* |
| KP072053 | *Epinephelus corallicola* |
| OP980557 | *Epinephelus polyphekadion* |
| KC845547 | *Cromileptes altivelis* |
| KC593375 | *Cromileptes altivelis* |
| OP980561 | *Cromileptes altivelis* |
| LC545417 | *Epinephelus aeneus* |
| OP035077 | *Mycteroperca bonaci* |
| OP056885 | *Mycteroperca microlepis* |
| PP032989 | *Mycteroperca acutirostris* |
| PP032972 | *Epinephelus marginatus* |
| KC816460 | *Epinephelus epistictus* |
| KC593373 | *Epinephelus epistictus* |
| KC593371 | *Triso dermopterus* |
| OP056896 | *Hyporthodus niveatus* |
| FJ594966 | *Hyporthodus septemfasciatus* |
| JX135579 | *Hyporthodus octofasciatus* |
| KC537759 | *Cephalopholis boenak* |
| KC593377 | *Cephalopholis argus* |
| KP833628 | *Aethaloperca rogaa* |
| KC593376 | *Aethaloperca rogaa* |
| PP032959 | *Cephalopholis fulva* |
| OQ420715 | *Cephalopholis taeniops* |
| MW560467 | *Cephalopholis leopardus* |
| MW423580 | *Cephalopholis miniata* |
| MW560468 | *Cephalopholis spiloparaea* |
| KC593378 | *Cephalopholis sonnerati* |
| MZ411547 | *Cephalopholis urodeta* |
| KU891818 | *Cephalopholis urodeta* |
| KJ469385 | *Cephalopholis sexmaculata* |
| MN688375 | *Plectropomus laevis* |
| KC262636 | *Plectropomus areolatus* |
| JQ420073 | *Plectropomus leopardus* |
| KJ101556 | *Plectropomus leopardus* |
| JQ420074 | *Plectropomus leopardus* |
| KJ101555 | *Plectropomus leopardus* |
| KC593370 | *Variola albimarginata* |
| NC_022138 | *Variola louti* |
| NC_019572 | *Perca flavescens* |
| NC_005254 | *Etheostoma radiosum* |
| MW689259 | *Toxotes chatareus* |
| NC_004406 | *Caranx melampygus* |
|  |  |

Table S2. Protein Codon Usage Analysis in the mitochondrial genome of *Epinephelus itajara.* It is listed amino acid identity, codon identity, and how often a particular codon is used in the mitochondrial genome.
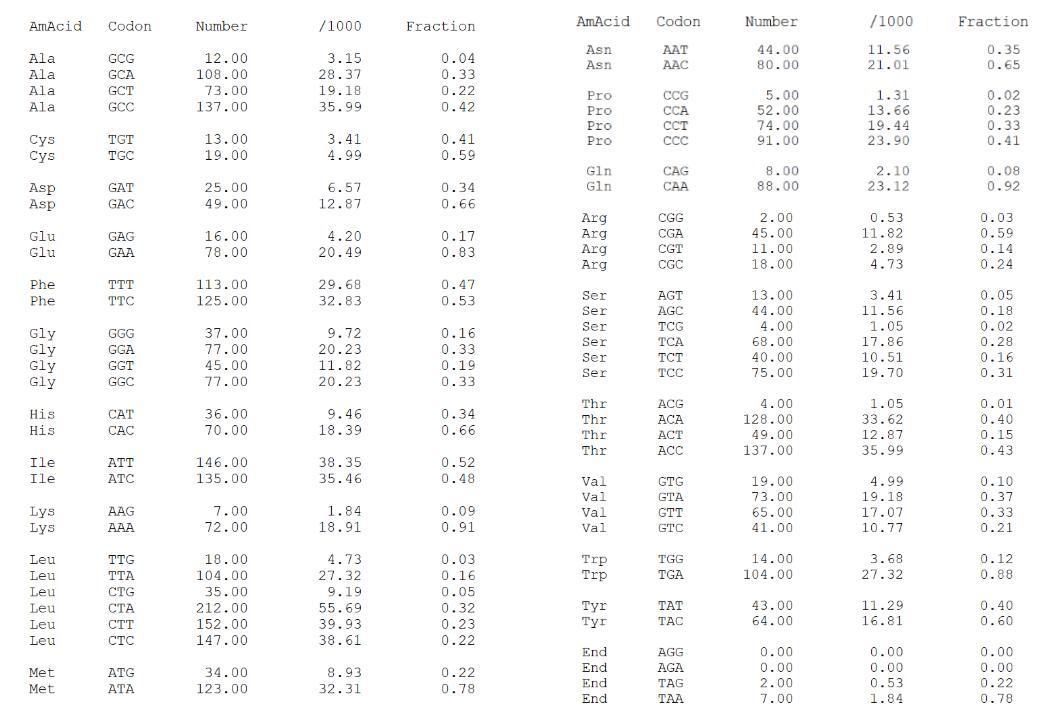


Table S3. Microsatellite repeats in the mitochondrial control region of *Epinephelus itajara*.


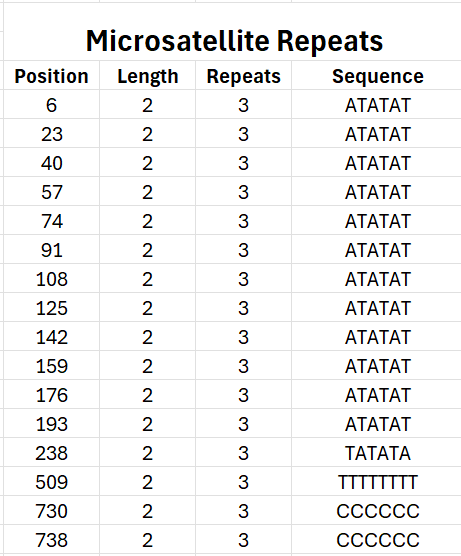


Table S4. Tandem repeats found in the mitochondrial control region of *Epinephelus itajara*. Information about the location (Indices), length (Period Size), how many times is repeated Copy number), and nucleotide use is shown.
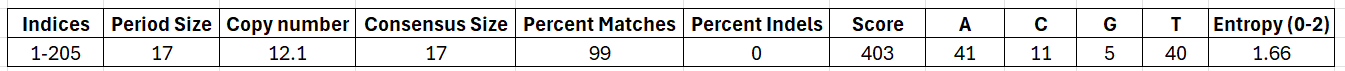

Supplement: Supplementary file 1 — Data S1. [file ECE3-15-e71795-s001.docx]
